# Identification of Novel Small Organic Compounds with Diverse Structures for the Induction of Epstein-Barr Virus (EBV) Lytic Cycle in EBV-Positive Epithelial Malignancies

**DOI:** 10.1371/journal.pone.0145994

**Published:** 2015-12-30

**Authors:** Chung King Choi, Dona N. Ho, Kwai Fung Hui, Richard Y. Kao, Alan K. S. Chiang

**Affiliations:** 1 Department of Paediatrics and Adolescent Medicine, Li Ka Shing Faculty of Medicine, The University of Hong Kong, Hong Kong SAR, China; 2 Department of Microbiology, Li Ka Shing Faculty of Medicine, The University of Hong Kong, Hong Kong SAR, China; 3 Center for Nasopharyngeal Carcinoma Research, The University of Hong Kong, Hong Kong SAR, China; Gustave Roussy, FRANCE

## Abstract

Phorbol esters, which are protein kinase C (PKC) activators, and histone deacetylase (HDAC) inhibitors, which cause enhanced acetylation of cellular proteins, are the main classes of chemical inducers of Epstein-Barr virus (EBV) lytic cycle in latently EBV-infected cells acting through the PKC pathway. Chemical inducers which induce EBV lytic cycle through alternative cellular pathways may aid in defining the mechanisms leading to lytic cycle reactivation and improve cells’ responsiveness towards lytic induction. We performed a phenotypic screening on a chemical library of 50,240 novel small organic compounds to identify novel class(es) of strong inducer(s) of EBV lytic cycle in gastric carcinoma (GC) and nasopharyngeal carcinoma (NPC) cells. Five hit compounds were selected after three successive rounds of increasingly stringent screening. All five compounds are structurally diverse from each other and distinct from phorbol esters or HDAC inhibitors. They neither cause hyperacetylation of histone proteins nor significant PKC activation at their working concentrations, suggesting that their biological mode of action are distinct from that of the known chemical inducers. Two of the five compounds with rapid lytic-inducing action were further studied for their mechanisms of induction of EBV lytic cycle. Unlike HDAC inhibitors, lytic induction by both compounds was not inhibited by rottlerin, a specific inhibitor of PKCδ. Interestingly, both compounds could cooperate with HDAC inhibitors to enhance EBV lytic cycle induction in EBV-positive epithelial cancer cells, paving way for the development of strategies to increase cells’ responsiveness towards lytic reactivation. One of the two compounds bears structural resemblance to iron chelators and the other strongly activates the MAPK pathways. These structurally diverse novel organic compounds may represent potential new classes of chemicals that can be used to investigate any alternative mechanism(s) leading to EBV lytic cycle reactivation from latency.

## Introduction

Epstein-Barr virus (EBV) is a ubiquitous gammaherpesvirus which infects over 90% of the adult population worldwide. Its acute infection sometimes causes infectious mononucleosis, though most of the time its infection is asymptomatic [1, 2]. EBV adopts a biphasic life cycle as other herpesviruses and persists in latencies in infected cells after initial infection, expressing only a limited number of viral proteins and transcripts. Reactivation of the latent virus into lytic cycle induces the expression of a temporally regulated cascade of approximately 80 lytic proteins. The reactivation of lytic cycle in latently-infected cells can be induced by a variety of agents, e.g. anti-immunoglobulin [3, 4], tumour growth factor β (TGF-β) [5, 6], and different groups of chemicals [7]. Histone deacetylase (HDAC) inhibitors [8–11] and phorbol esters [12–14] are the major classes of chemical lytic inducers reported thus far.

EBV has been suggested to underlie the development of various lymphoid and epithelial cancers for their persistence in the infected cells, e.g. Burkitt lymphomas (BL), Hodgkin lymphomas, nasopharyngeal carcinoma (NPC), gastric carcinoma (GC), etc. Although EBV is present in latent states in these tumours, substantiating the importance of latent infection in oncogenesis, accumulating evidence has also pointed to the possible contribution of EBV lytic reactivation towards tumour development. Fang *et al*. [15, 16] reported that repeated lytic reactivation by chemical lytic inducers enhanced genome instability in NPC cells and promoted tumour progression in NPC mouse xenograft models. Hong *et al*. showed that for lymphoblastoid cell lines (LCLs), those with immediately-early gene-deleted EBV genomes incapable of going into lytic cycle produced lower level of the angiogenesis factor vascular endothelial growth factor (VEGF) [17], and less capable of forming tumours in a SCID mice model [18]. Despite such implication in disease progression, the presence of EBV in these tumours presents a unique target that can be exploited for treating EBV-positive malignancies. Lytic induction therapy, in which a combination of lytic inducer and antiviral drug is employed for the specific killing of EBV-positive tumour cells, is one of such strategies [9, 19–21]. Antiviral drugs for herpesviruses, e.g. acyclovir and ganciclovir, are originally non-cytotoxic to these EBV-positive tumours since the viruses in latent states do not express the viral protein kinase BGLF4 required to convert these drugs to an active cytotoxic form [22]. However, when the latent viruses are induced into lytic cycle and express BGLF4, the drugs can be converted into the cytotoxic form by the viral kinases and kill the cancer cells. This method has been demonstrated to be effective in *in vitro* assays and mouse models [19, 23, 24], and has been entering clinical trials [9, 20, 21]. One limiting factor of the effectiveness of this therapeutic strategy is the cells’ responsiveness to EBV lytic induction. *In vitro*, the efficacy of these chemical lytic inducers is highly dependent on cellular background [10, 25–28], and there remains a cell population refractory to lytic induction upon the application of any agents. Increasing the cells’ responsiveness is thus vital to the development of this novel therapeutic strategy.

To this end, a lot of efforts have been dedicated to studying the mechanisms EBV lytic reactivation *in vitro* with EBV-positive B cells or epithelial cells as models. To date, a number of kinase pathways, including the phosphatidylinositol 3-kinase (PI3K) [5], mitogen-activated protein kinases (MAPKs) [29–32], protein kinase C (PKC) [8, 14, 33], and ataxia telangiectasia mutated (ATM) kinase [7] pathways, have been reported to mediate lytic reactivation by lytic inducing stimuli in different cell backgrounds. In particular, the chemical inducers phorbol ester 12-*O*-tetradecanoylphorbol-13-acetate (TPA) and HDAC inhibitors have been reported to act through PKC to activate the transcription of the immediately-early (IE) protein Zta [8, 14, 33, 34], whose expression alone is sufficient to trigger the switch from latent to lytic cycle [35, 36]. Nevertheless, conflicting evidence suggests that activation of PKC is neither sufficient nor necessary for lytic induction by HDAC inhibitors [28]. More thorough understanding of the molecular events leading to lytic reactivation is vital to devising strategies to achieve higher responsiveness of any chemical intervention.

We conceive that the identification of novel organic molecules to induce EBV lytic cycle would add to the pool of stimulants for the *in vitro* study of mechanisms leading to lytic reactivation, we performed a high-throughput screening with more than 50,000 small novel organic molecules, and identified 5 structurally diverse compounds that can potently induce EBV lytic cycle in EBV-positive epithelial malignancies. Compared to HDAC inhibitors and phorbol esters, these novel compounds are structurally distinct and do not seem to possess similar biological activities. They can also act in concert with HDAC inhibitors to synergistically induce lytic cycle. Thus we consider them as attractive targets for further study into the mechanism of action of lytic reactivation and as lead compounds to uncover new classes of chemical EBV lytic inducers.

## Materials and Methods

### Cell Culture

AGS is an EBV-negative gastric carcinoma (GC) cell line [37], and AGS-BX1 was generated by introducing an recombinant Akata EBV genome into AGS cells (gifts from Prof. Lindsey M. Hutt-Fletcher, Louisiana State University, LA) [38]. HONE1-EBV was generated by introducing a recombinant Akata EBV genome into the EBV-negative NPC cell line HONE1 (gift from Prof. GSW Tsao) [39]. These three cell lines were cultured as previously described unless otherwise specified [10, 40]. Both AGS-BX1 and HONE1-EBV cells contain a green fluorescent protein (GFP) open reading frame in the EBV genome. NA is a Taiwanese EBV-positive NPC cell line obtained by infecting the EBV-negative TW01 cell line with an Akata EBV genome carrying neomycin-resistant gene (gift from Prof. Ching-Hwa Tsai and Prof. Jen-Yang Chen, National Taiwan University, Taiwan). It was maintained in RPMI-1640 (Life Technologies) supplemented with 10% heat-inactivated fetal bovine serum (FBS; Life Technologies) and 500μg/ml G418 (Merck KGaA, Damstadt, Germany) [41]. SNU-719 [42, 43] (Korean Cell Line Bank, No. 00719) and YCCEL1 [44] (gift from Prof. Qian Tao, The Chinese University of Hong Kong, Hong Kong) are EBV-positive GC cell lines harbouring native EBV genomes. C666-1 is an EBV-positive NPC cell line harboring native EBV genomes [45]. Daudi is an EBV-positive Burkitt lymphoma cell line that is permissive to EBV superinfection [10]. These four cell lines were maintained in RPMI-1640 supplemented with 10% heat-inactivated FBS.

### Chemicals

The small organic molecules for primary high-throughput screening, secondary screening, tertiary screening and subsequent characterization were all obtained from ChemBridge Corp. (San Diego, CA, USA). Suberoylanilide hydroxamic acid (SAHA) was obtained from Cayman Chemical (Ann Arbor, MI, USA). Romidepsin and Ku-55933 (Ku) were obtained from Selleck Chemicals (Houston, TX, USA). LY294002 (LY), PD98059 (PD), SP600125 (SP), SB202190 (SB) and rottlerin were purchased from Merck (Merck KGaA, Damstadt, Germany).

### Assay for Cell Proliferation

Cell proliferation was assayed by 3-(4,5-dimethylthiazol-2-yl)-2,5-diphenyltetrazolium bromide (MTT). EBV-positive AGS-BX1 cells and EBV-negative AGS cells (2×10^4^ cells/well) were seeded in 96-well cell culture plates and incubated overnight at 37°C, 5% CO_2_. Cells were either untreated or treated with various concentrations of the lytic-inducing compounds for 48h in triplicates. 10μl of MTT solution at 5mg/ml (Life Technologies) was then added to each well containing 100μl cells and the cells were then incubated at 37°C, 5% CO_2_ for 4h. 100μl 10% SDS (w/v)/0.1M HCl was added to each well to lyse the cells and dissolve the resulted formazan crystals. Measurement of absorbance at 570nm was carried out after overnight incubation of the plates at 37°C with iMark Microplate Absorbance Reader (Bio-Rad, Hercules, CA). The results shown were obtained from 5 independent experiments and standard errors were shown as error bars.

### Western Blot Analysis

Cells were grown to 70% confluence before treating with chemicals. At specified treatment time, cells were harvested and the cell pellet was washed with phosphate buffered saline (PBS, Sigma-Aldrich) once. Proteins were extracted from the cells and separated by 10% acrylamide gels as previously described [10]. EBV proteins were detected as previously described [10, 40]. Histone acetylation was detected with anti-acetylated-histone 3 (H3) rabbit polyclonal antibodies (1:2000, Millipore). Activation of the kinase pathways was detected with anti-p-p38 MAPK, anti-p-JNK, anti-p-PKCδ (1:1000) and anti-p-ATM (1:500) rabbit polyclonal antibodies (Cell Signaling Technology). Expression of human α-tubulin or β-actin was detected with anti-α-tubulin (1:5000) or anti-β-actin (1:10000) antibodies (Sigma-Aldrich) as loading controls.

### Quantitative PCR Assay

AGS-BX1 or NA cells grown to 70% confluence were treated with lytic-inducing compounds at doses nearest to inhibiting 50% cell proliferation for 48h. Cells were harvested and washed once with PBS. DNA was extracted from the cells and quantitative PCR was performed as previously described [10]. The results were presented as the number of EBV genome copies per cell for triplicate wells in 96-well plates for each treatment condition.

### Immunocytochemistry

AGS-BX1 cells were seeded on cover slips coated with 0.1% gelatin in 24-well cell culture plates. Cells grown to 70% confluence were treated with the lytic-inducing compounds for 3 days, then fixed and stained with anti-Zta mouse monoclonal antibody [46] (gift from Prof. P. Farrell, Imperial College London, UK) as previously described [10]. The nuclei of cells were visualised with 4’,6’-diamidino-2-phenylidole (DAPI, Roche).

### Flow Cytometry Analysis for Percentage Cells Induced into Lytic Cycle

AGS-BX1 cells were allowed to grow to 70% confluence before treatment with the lytic cycle inducing compounds for 3 days. One million cells were collected for each condition and washed once with PBS, followed by fixation and permeabilisation by the FACS fixation solution and FACS permeabilizing solution (BD Biosciences) for 30min each. Cells going into lytic cycle were then stained by the anti-Zta mouse monoclonal antibody [46] (1:50 in 5% normal goat serum) overnight at 4°C followed by Alexa Fluor 647 F(ab’)2 fragment of goat anti-mouse IgG (1:500; Life Technologies) at 37°C for 1h. The stained cells were then subject to analysis by flow cytometry (LSRII, BD Biosciences) and the Zta-positive population were taken as the population of cells induced into lytic cycle.

### EBV Infection Assay

AGS-BX1 cells at 70% confluence were treated with the lytic-inducing compounds for 5 days or untreated. The culture supernatants were collected and centrifuged, then filtered with 0.45μm syringe filters (Sartorius Stedim Biotech, Goettingen, Germany). The filtered supernatants were used to superinfect Daudi cells as previously described [10]. The Daudi cells were analysed by flow cytometry (LSRII, BD Biosciences) for GFP expression to give an estimation of the level of infectious virus particles released into the culture supernatants upon compound treatment.

### Primary High-Throughput Screening (HTS)

The primary HTS was performed in a previously described automated Beckman Coulter Core System (Fullerton) integrated with a Kendro robotics CO_2_ incubator (Thermo Scientific) at the Department of Microbiology, LKS Faculty of Medicine, The University of Hong Kong [47]. The primary screening library comprised 50,240 structurally diverse small organic molecules dissolved in dimethyl sulfoxide (DMSO) from the DIVERSet screening library from ChemBridge Corp. (San Diego, CA, USA). These compounds were dispensed at a final concentration of 20μg/ml in 384-well microliter plates in triplicate, to which AGS-BX1 cells were added at 6000 cells per well in 25μl Ham’s F12 (Life Technologies) supplemented with 5% heat-inactivated FBS (Life Technologies). Two columns of untreated cells as negative controls and 5μM SAHA-treated cells as positive controls were included in each plate. After 3-day incubation at 37°C with 5% CO_2_, green fluorescent signal was read at 535nm and recorded in DTX 880 multimode detector (Beckman Coulter). The compounds which induced an increase in green fluorescence signal larger than 1.5 fold of the negative control were selected as hits of the primary HTS.

### Secondary Screening

The secondary screening, the cytoblot assay, was performed with two cell lines, AGS-BX1 and NA. 30,000 cells of AGS-BX1 or 10,000 cells of NA were seeded per well in white opaque 96-well plates (Greiner Bio-One) overnight before the addition of selected compounds. The selected compounds were assayed at two concentrations, 1μg/ml and 10μg/ml, in duplicates. After 24h incubation with the compounds at 37°C with 5% CO_2_, the induction of EBV lytic cycle was detected by probing for the expression of the EBV immediately-early (IE) protein Zta in cells using enhanced chemiluminescence after fixing the cells on plate with 4% paraformaldehyde in PBS for 15min and blocking with 0.5% bovine serum albumin (BSA, Sigma-Aldrich), 0.1% Triton X-100 in PBS for 1h. The cells after fixation and blocking were incubated with anti-Zta monoclonal antibody [46] at 1:600 overnight at 4°C, after which horseradish peroxidase-conjugated goat anti-mouse IgG at 1:1000 dilution were bound for 1h at room temperature and the luminescence signal were measured by a standard plate reader after the addition of chemiluminescence substrate (Pierce, Thermo Scientific). A column of untreated cells and 5μM SAHA-treated cells were included as the negative and positive controls respectively. The luminescent signals were normalised to the negative controls on the same plate, and compounds with readings from both of the duplicate wells passing the cutoff value were ranked based on the normalised reading, regardless of the concentrations used. Top 40 hits in each cell line comprise the list of candidate compounds for next round of selection.

### Tertiary Screening

The cytotoxicity of each selected compound in AGS-BX1 and NA cells was assayed by the MTT assay at 5μg/ml, 10 μg/ml, 20 μg/ml and 40 μg/ml in duplicate after 48h of treatment. The concentrations inhibited/or closest to inhibiting 50% cell proliferation were taken as IC_50_ and used in subsequent assays in the tertiary screening.

More functional assays were employed at this stage to confirm the EBV lytic-inducing ability of the selected compounds. To compare the strength of lytic induction by the selected hit compounds, AGS-BX1 or NA cells grown to 70% confluence were treated with the selected compounds at their IC_50_ determined at 48h and harvested to examine the expression of various lytic proteins, the IE proteins Zta and Rta, and the early protein EA-D (BMRF1) by Western blot analysis. 22 compounds with strong expression of IE and early EBV lytic proteins were selected for further comparison, including an additional round of Western blotting and quantitative PCR to examine the replication of viral genome inside cells at 48h post-treatment.

## Results

### Phenotype-Based High-Throughput Screening (HTS) Identified 5 Novel Organic Molecules that Can Potently Induce EBV Lytic Cycle in EBV-Positive Epithelial Cancer Cells

**Fig 1:**
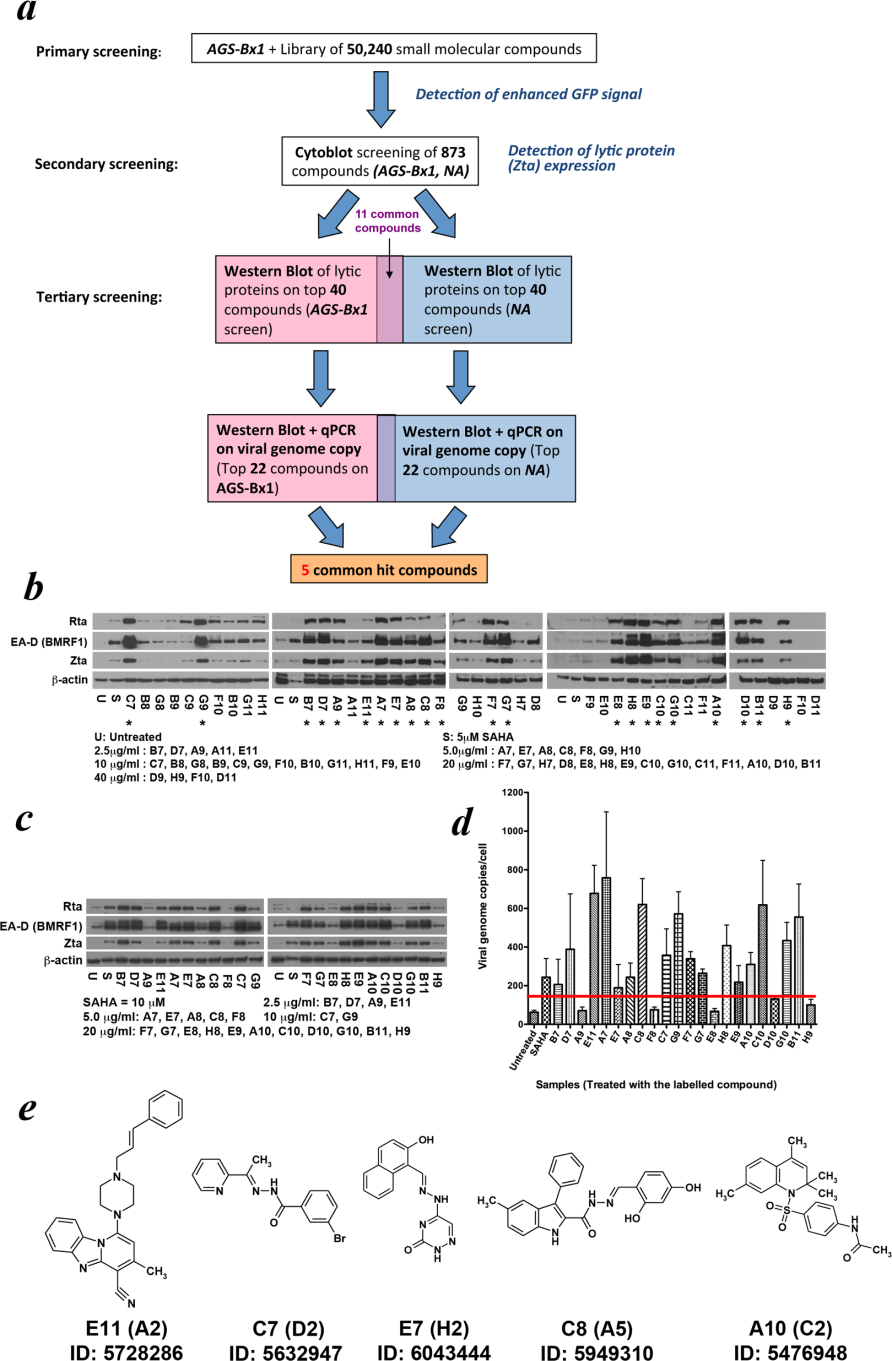
High-throughput screening of a chemical library of 50,240 organic compounds for EBV lytic induction in EBV-positive epithelial malignancies. (a) Flow chart of the screening process. (b) Expression of EBV immediately-early (IE) lytic proteins, Zta, Rta, and early protein EA-D (BMRF1) in AGS-BX1 cells 48h post-treatment by the top 40 compounds in tertiary screening. The concentrations used were the approximate half inhibitory concentration (IC_50_) for cell proliferation. The 22 compounds with an asterisk (*) below their code were selected for further comparison of lytic protein expression and viral genome replication upon addition to the cells. (c) & (d) Expression of EBV IE and early proteins and replication of viral genome 48h post-treatment induced by the selected 22 compounds on AGS-BX1 cells in tertiary screening. (e) Structure of the 5 hit compounds identified in the HTS. The compound codes used for AGS-BX1 cells (NA cells), as well as the compound IDs at the ChemBridge Corp. catalogue are given below each structure.

AGS-BX1, an EBV-positive GC cell line, contains a GFP open reading frame in the BXLF1 gene in its EBV genome [38] and expresses GFP upon lytic induction with the fluorescent signal strength in approximate correlation to the strength of lytic induction [10], providing a quantifiable readout for HTS. To identify novel organic molecules that can effectively reactivate EBV lytic cycle, we screened a library of 50,240 small organic molecules from the DIVERSet screening library (ChemBridge Corp.) with AGS-BX1 cells on the HTS platform established at the Department of Microbiology at the University of Hong Kong, using GFP as readout (refer to Fig 1A for an outline of the screening process). The molecules in the library were assayed for 3 days at 20μg/ml and 873 compounds displaying fluorescent signal larger than 1.5 fold of the untreated controls were selected. The secondary screen, the Cytoblot assay, utilized NA, a Taiwanese EBV-positive NPC cell line, in addition to AGS-BX1. In this round of screening, the lytic induction strength was assayed by detecting the expression of immediately-early (IE) viral protein Zta in cells by chemiluminescence after 24h incubation with the test compounds, and this further reduced the number of hit molecules to 40 in each cell line with the strongest expression of Zta. Among the identified molecules, there were 11 overlaps for the two cell lines. To validate the lytic inducing ability of these selected molecules, functional assays were performed with these molecules at the concentrations that inhibited approximately 50% cell proliferation (IC_50_) at the tertiary screening stage in both AGS-BX1 and NA cells, the two cell lines used in the secondary screen. The 40 hits in each cell line were first tested with Western blotting for their ability to trigger the expression of multiple EBV lytic proteins, including the IE proteins Zta and Rta, and the early protein EA-D (BMRF1) (Fig 1B and S1A Fig). 22 molecules from each cell line that induced strong expression of these proteins were selected for further comparison (Fig 1C and S1B Fig). The 22 molecules on each cell line were tested again with Western blotting for lytic protein expression, as well as with quantitative PCR for viral genome replication (Fig 1D and S1C Fig) The molecules which induced strong expression of IE and early lytic proteins, as well as viral genome replication in both cell lines were selected as hits of the screening. By these criteria, 5 molecules were chosen based on their potent ability to induce lytic cycle on both AGS-BX1 and NA cells (Fig 1E).

### The Newly Identified Compounds Induce Lytic Cycle in a Dose-Dependent, Time-Dependent and Cell Line-Dependent Manner

**Fig 2:**
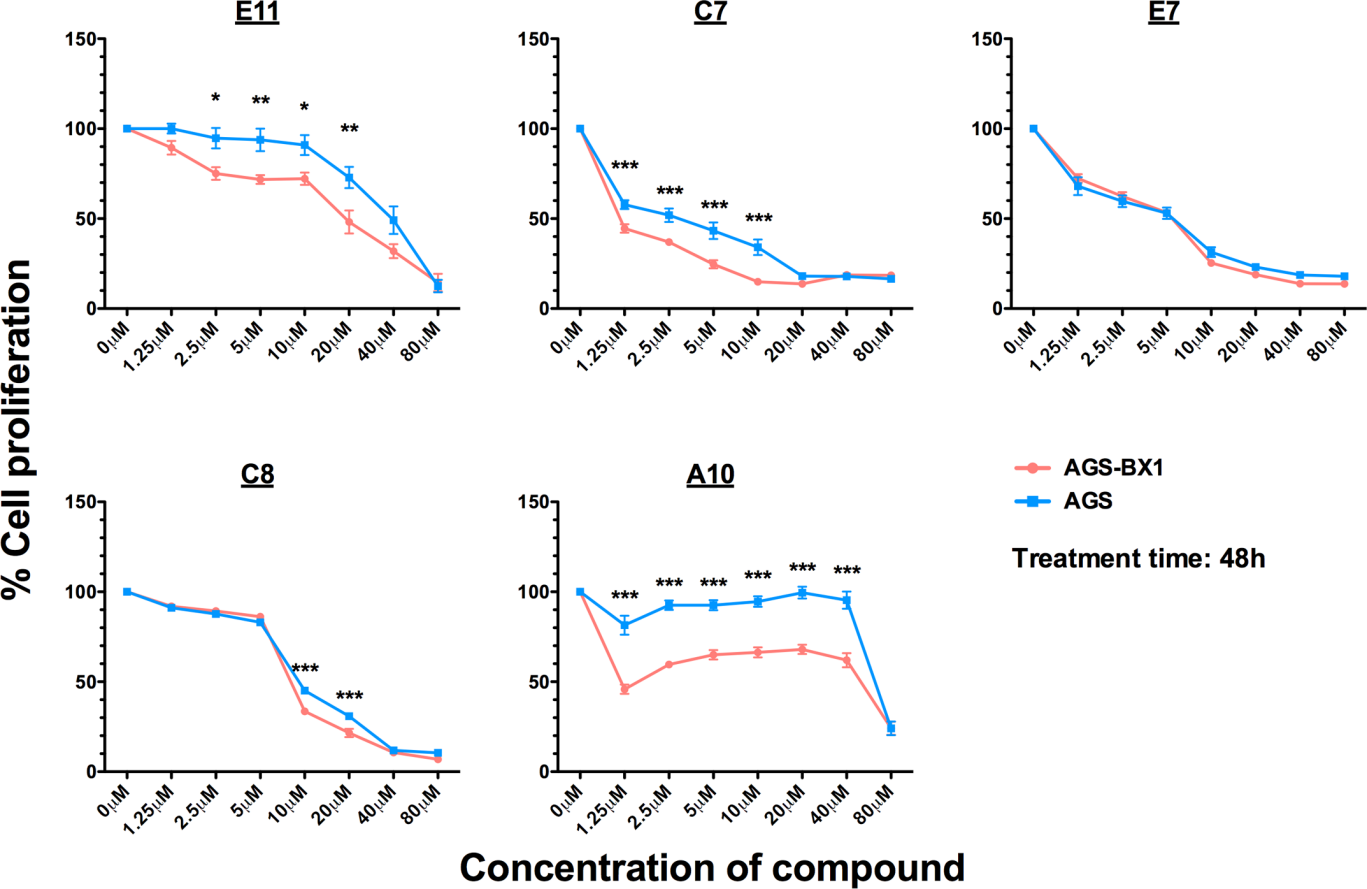
Cytotoxicity of the hit compounds in AGS and AGS-BX1 cells assayed by MTT at 48h post-treatment. AGS or AGS-BX1 cells were incubated with various concentrations of the hit compounds for 48h and the cytotoxicity was assayed by MTT as described in the Materials and Methods section. Results are expressed as percentages of the cell proliferation of the untreated cells and data from 5 independent experiments are presented. Standard errors are shown in error bars. One-way ANOVA and Bonferroni post-test were performed in GraphPad Prism 5 to compare the difference in cell proliferation between the EBV-positive AGS-BX1 and EBV-negative AGS cells (* p<0.05, ** p<0.01, ***p<0.001).

**Fig 3:**
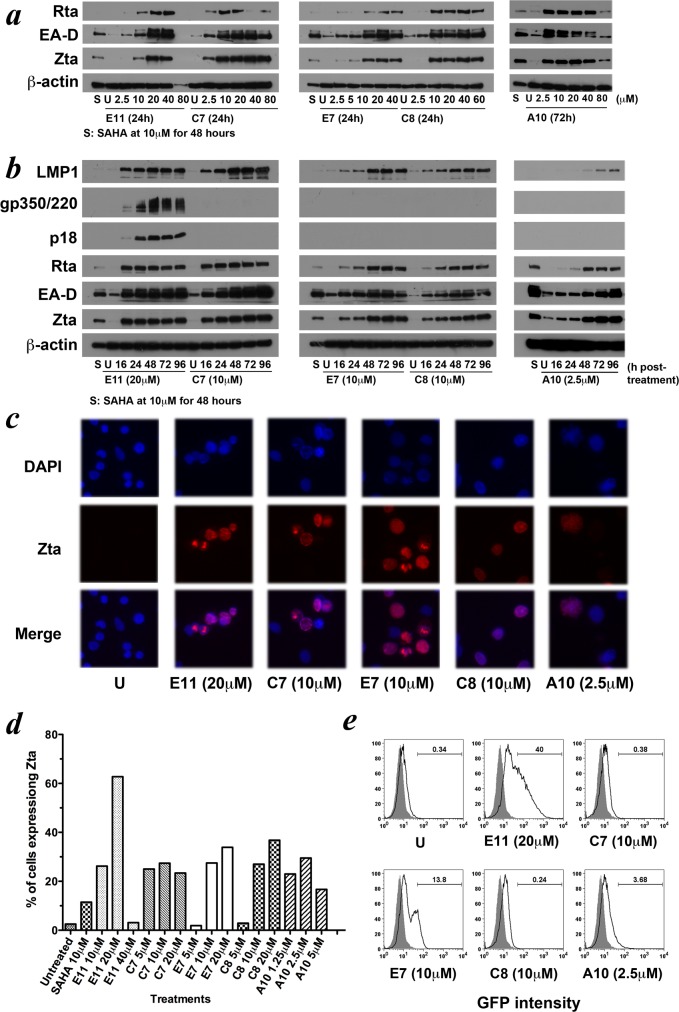
EBV lytic induction of the 5 hit compounds in the EBV-positive GC cells. AGS-BX1 cells were treated with (a) various concentrations of the hit compounds or (b) the hit compounds at the concentrations which maximally induced lytic cycle (optimal concentrations) at various time points. Expression of various EBV lytic proteins were analysed with Western blotting with cellular β-actin as a loading control. (c) AGS-BX1 cells were treated with the hit compounds at their optimal concentrations for 72h. The expression of Zta was visualized by immunofluorescent staining (red; middle panel) and the nuclei were visualized by DAPI (blue; top panel). (d) AGS-BX1 cells were treated with the hit compounds at their optimal concentrations for 72h. The percentage of cells induced into lytic cycle, i.e. expressing the IE protein Zta, was quantified by flow cytometry. (e) Production of infectious virions in AGS-BX1 cells after treatment with the hit compounds. AGS-Bx1 cells, which are capable of producing GFP-tagged EBV virions, were first treated by the hit compounds for 5 days. The supernatants containing the virions produced were then collected and incubated with Daudi cells, which are susceptible to superinfection of EBV. Percentage of superinfected Daudi cells, which were GFP-positive, were shown on the graph. The grey area represented unstained Daudi cells incubated with culture medium only.

For detailed characterization of these newly identified compounds, we tested their cytotoxicity in EBV-positive and EBV-negative paired cell lines. We also tested the dose response, kinetics response of the compounds, the percentage of cells being induced into lytic cycle, and the ability to induce the production of infectious virus in AGS-BX1 cells upon treatment by each compound. Of the 5 compounds identified, 4 (compounds coded E11, C7, C8 and A10) displayed significantly higher toxicity to the EBV-positive cell line AGS-BX1 than the EBV-negative counterpart AGS at the lytic-inducing concentrations, demonstrating EBV-specific killing of these compounds (Fig 2). The 5 compounds could all induce dose-dependent expression of EBV viral lytic proteins at micromolar concentrations on AGS-BX1 cells, with maximal induction observed at doses ranging from 2.5 to 20μM (Fig 3A). The lytic induction kinetics varied between compounds, the fastest of which being the compounds coded E11 and C7, with the expression of IE proteins Zta, Rta, and early EBV lytic protein BMRF1 peaking at 24h post-treatment. Other compounds exhibited slower lytic induction kinetics, with the expression of Zta, Rta, and BMRF1 peaking at 48-72h post-treatment (Fig 3B). Interestingly, further time point experiments revealed that the expression of the master switch protein of lytic cycle, Zta, was detected even 15min after the incubation of E11 and C7 with the AGS-BX1 cells (S2 Fig). Such fast action and high potency make them attractive targets for investigation into their mechanism of action. Although all 5 compounds could potently induce the expression of IE and early EBV lytic proteins, not all induced the expression of late proteins. Among the 5, only E11 consistently induced the expression of late proteins, e.g. VCA-p18 and/or gp350/220 in AGS-BX1, HONE1-EBV and YCCEL1 cells (Fig 3B and S3 Fig). The expression of late proteins correlated closely with the production of infectious viral particles in the EBV infection assay. In particular, after a 5-day incubation with the compound triggered the most expression of late proteins E11, the filtered culture supernatant from AGS-BX1 cells infected 40% of the Daudi cells in the EBV infection assay (Fig 3F)–the most among the 5 hit compounds. Immunofluorescent staining of AGS-BX1 cells after 3-day incubation with the compounds revealed that most of the Zta proteins were localised within the nuclei of cells (Fig 3D), consistent with their role as a transcription transactivator and replication-associated factor [3, 48, 49]. We quantified the percentage of cells expressing Zta by flow cytometry to obtain an estimation of cells going into lytic cycle. On average the Zta-positive population approximately ranged from 20% to 40%, with E11 inducing the expression of Zta in up to 65% of cells (Fig 3E).

**Fig 4:**
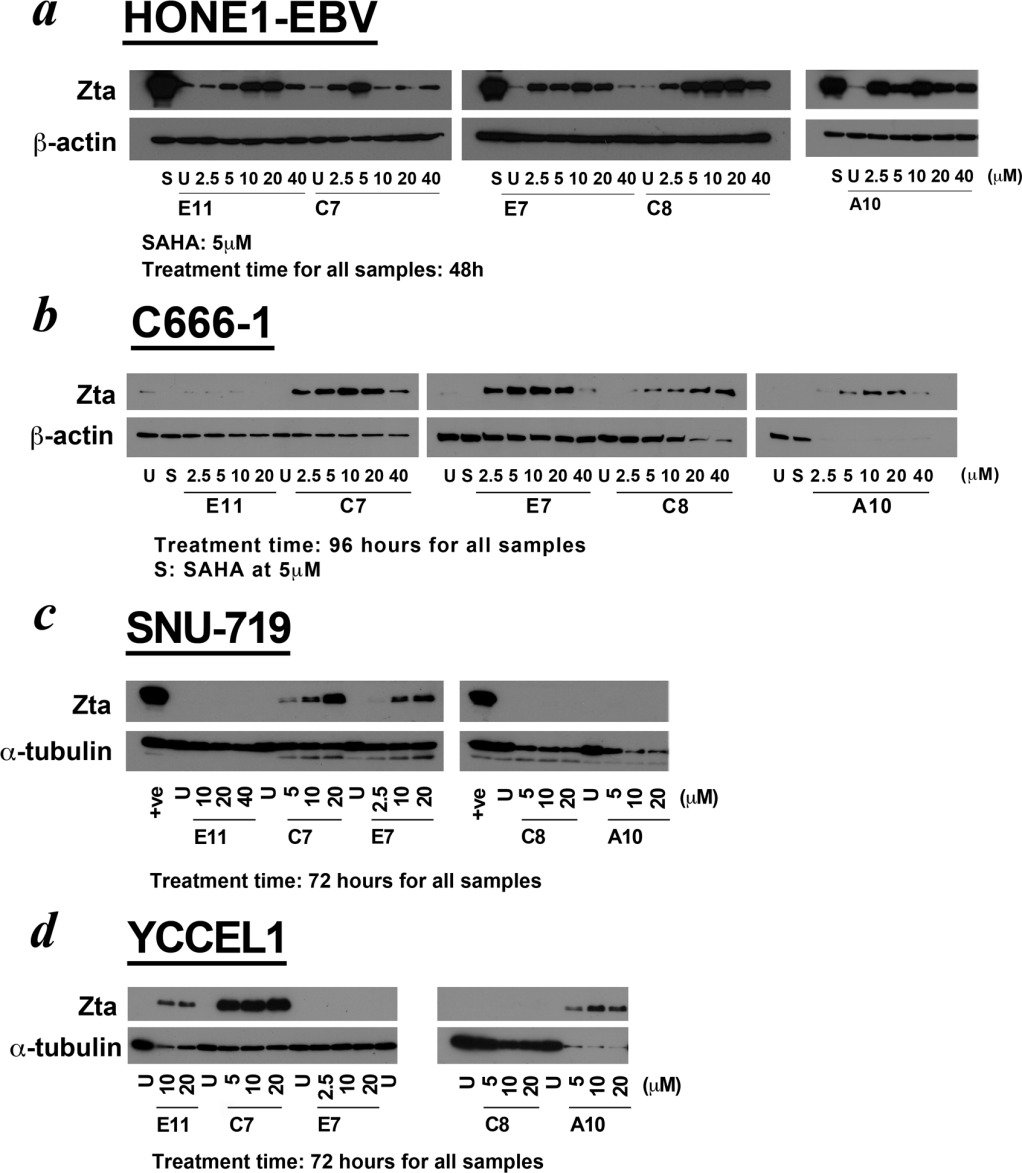
EBV lytic induction of the 5 hit compounds in several EBV-positive epithelial cell lines. **(**a) HONE1-EBV cells, (b) C666-1 cells, (c) SNU-719 cells, and (d) YCCEL1 cells were treated with the hit compounds at various concentrations for the specified period. HONE1-EBV cells contain a recombinant Akata genome while C666-1 cells, SNU-719 cells and YCCEL1 cells all contain native EBV genomes. The expression of viral IE protein Zta after treatment was detected by western blotting. The compound coded C7 could induce EBV lytic cycle in all the cell lines tested.

We further tested these newly discovered compounds in other EBV-positive epithelial cancer lines (Fig 4), as the action of most common lytic inducers has been reported to be cell line-dependent [10, 25]. All the five compounds could reactivate lytic cycle in HONE1-EBV cells, which is an NPC cell line with recombinant EBV genome. Not all compounds could reactivate lytic cycle in the cell lines with native EBV genomes. 4 out of the 5 compounds reactivated EBV lytic cycle in C666-1 cells, the only currently available authentic NPC cell line with native EBV genomes. However, compound E11, which induced lytic cycle strongly in AGS-BX1 cells, could not reactivate lytic cycle in C666-1 cells. For GC cell lines harbouring native EBV genome, only compounds C7 and E7 reactivated lytic cycle in SNU-719 cells, and only E11, C7 and A10 could reactivate lytic cycle in YCCEL1 cells. Although the other 4 compounds exhibit cell line-dependent induction, compound C7 could induce EBV lytic cycle in all the EBV-positive epithelial cancer cell lines tested at micromolar concentrations.

### The Newly Identified Inducers Are Structurally and Biologically Distinct from Classical Lytic Inducers

**Fig 5:**
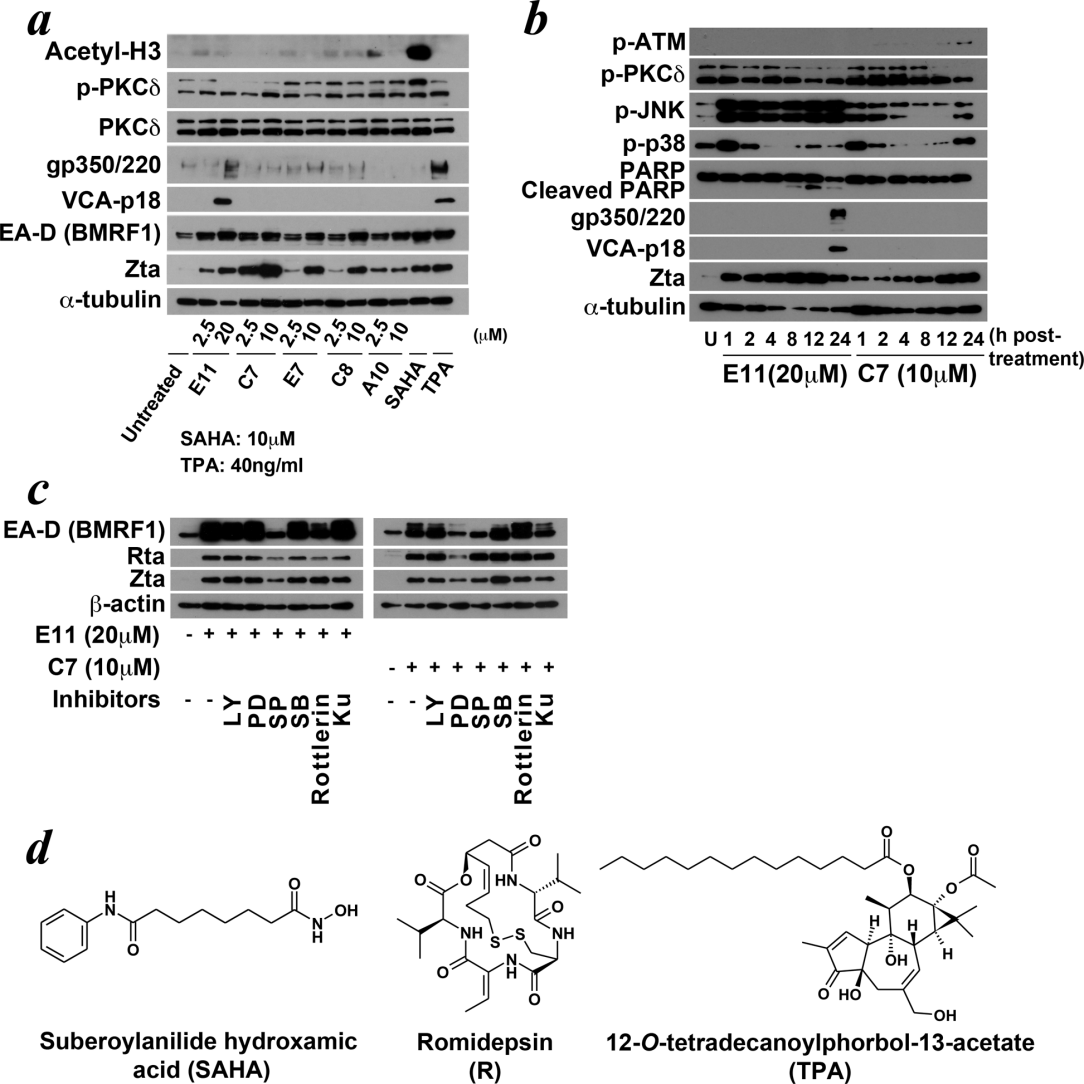
Difference in structures and biological activities between hit compounds and classical lytic inducers. (a) AGS-BX1 cells were treated with the hit compounds for 24h. Hyperacetylation of histone proteins and PKCδ phosphorylation were detected by Western blotting. No significant increase in the level of acetylated histone 3 (Acetyl-H3) and phosphorylated PKCδ was observed. (b) AGS-BX1 cells were treated by compound E11 or C7 for 1, 2, 4, 8, 12 24h. Changes in the phosphorylation of JNK and p38 MAPK were examined in conjunction with changes in the expression level of EBV proteins. JNK phosphorylation increased and sustained during the treatment period while level of phosphorylated p38 MAPK fluctuated across the treatment period. (c) AGS-BX1 cells were pre-treated with specific inhibitors of PI3K (LY294002, 15 μM, LY), MEK (PD98059, 50μM, PD), JNK (SP600125, 50μM, SP), p38 MAPK (SB202190, 20μM, SB), PKCδ (Rottlerin, 10μM) and ATM (KU-55933, 10μM, Ku) kinases for 1h before the addition of E11 and C7. Cells were harvest after 24h for examination of lytic induction by Western blotting. Lytic induction by E11 was significantly inhibited by SP, the specific JNK inhibitor while both PD (MEK inhibitor) and SP affected the induction by C7. (d) Structure of common HDAC inhibitors, SAHA and romidepsin, and the phorbol ester TPA, used for lytic induction of EBV. The structure of the newly identified compounds differs greatly from the known lytic inducers.

We compared the structures of the 5 novel compounds (Fig 1E) with each other and with those of the classical lytic inducers, the HDAC inhibitors and the phorbol ester TPA (Fig 5D). Except for the common possession of a hydrazone bridge for C7 and C8, the other hit compounds all possess distinct chemical structures. They are, too, structurally different from the HDAC inhibitors and phorbol esters. We also tried to compare the biological activities of the new inducers to HDAC inhibitors and phorbol ester. HDAC inhibitors, upon administration to the cells, caused rapid and sustained hyperacetylation of histone proteins [23]. As shown in Fig 5A, the newly identified compounds did not cause hyperacetylation of the histone 3 protein (Acetyl-H3), which is a marker for global histone hyperacetylation, after 24h incubation with AGS-BX1 cells as by SAHA. They did not lead to significant increase in protein kinase C δ (PKCδ) phosphorylation. We further investigated the biological effects of 2 of the 5 compounds, E11, which induced the largest proportion of AGS-BX1 cells into lytic cycle, and C7, which could induce all the cell lines tested into lytic cycle, in greater detail. We incubated these two compounds with AGS-BX1 cells for 1h, 2h, 4h, 8h, 12h and 24h respectively at the respective concentrations that maximally induce lytic cycle, and probed for the changes in several major kinase signalling pathways previously associated with EBV lytic cycle reactivation (Fig 5B). We observed strong and sustained phosphorylation of c-Jun N-terminal kinase (JNK) after treatment with E11 from 1h to 24h post-treatment, which was concurrent with the increase in protein level of Zta. After we treated the cells with C7, there was also an increase in phosphorylation of JNK at a level lower than that caused by E11 throughout the same treatment period. However, romidepsin, an HDAC inhibitor, did not increase the phosphorylation of JNK after 24h of treatment (S4 Fig). Apart from sustained increase in JNK phosphorylation, we also observed fluctuations in the level of phosphorylation of p38 MAPK kinase. Upon treatment of both compounds at 1h post-treatment, the level of p38 MAPK phosphorylation increased. It then went down at 2h post-treatment and remained low afterwards for E11, while the level went down from 2h to 12h then increased again at 24h post-treatment for C7. To test if the activation of these kinase pathways mediated EBV lytic reactivation, we tried to block these pathways with specific chemical pathway blockers of PI3K, MAPK/Erk kinase (MEK), JNK, p38 MAPK, PKCδ and ATM, which have been previously associated with EBV lytic reactivation [5, 8, 14, 29–33] (Fig 5C). We observed that only SP600125, a specific chemical inhibitor of JNK, weakened the expression of Zta, Rta, and EA-D (BMRF1) significantly upon the treatment by compound E11. For compound C7, more than one inhibitor appeared to be able to counteract the induction, though the effect being more partial. PD98095, the specific inhibitor of MEK, consistently reduced the expression of Rta, and SP600125 constantly reduced the expression of Zta and EA-D (BMRF1), suggesting that the induction of C7 might not be mainly mediated through a single pathway but two or more pathways. We have separately shown that only rottlerin, the specific inhibitor of PKCδ, was able to abrogate the lytic induction by the HDAC inhibitors romidepsin [23] and SAHA (S5 Fig). In comparison, compounds E11 and C7 produced substantially different response patterns upon the co-incubation with these specific inhibitors, further substantiating their difference in mechanism of action with the typical lytic inducers like HDAC inhibitors.

### The Novel Inducers Cooperated with HDAC Inhibitors to Induce EBV Lytic Cycle

**Fig 6:**
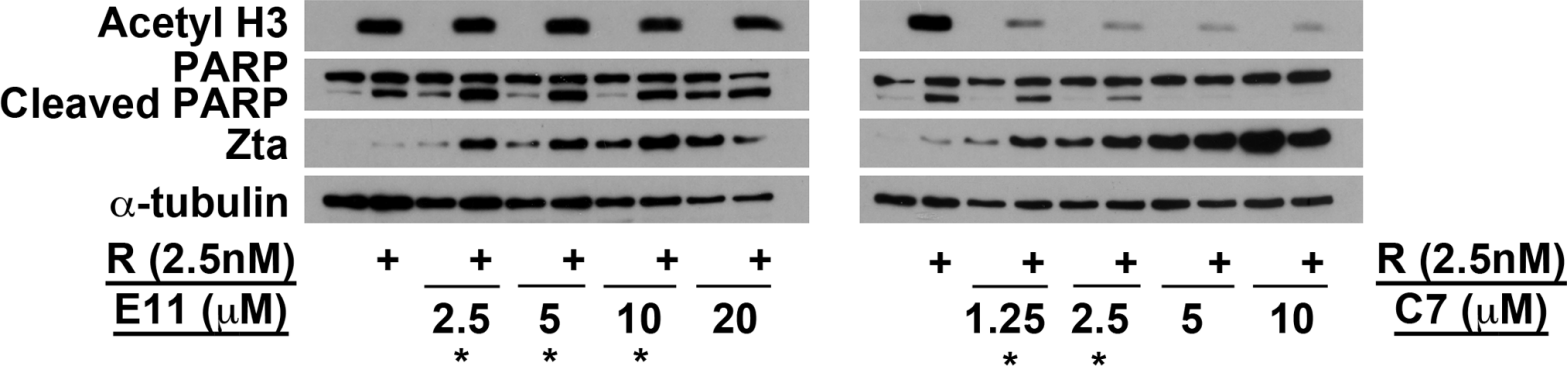
Enhanced induction of EBV lytic cycle by the combination of hit compounds and the HDAC inhibitor romidepsin. AGS-BX1 cells were treated with 2.5nM romidepsin (R) and various concentrations of E11 or C7 for 24h. Expression of viral IE protein Zta was detected by Western blotting to estimate the magnitude of lytic induction. The combinations with an asterisk (*) are the concentrations at which enhanced induction was observed.

The combination of two EBV lytic inducers does not necessarily reactivate EBV lytic cycle to a greater magnitude. We have previously reported that combining bortezomib and SAHA, both being reported to induce EBV lytic cycle on their own [10, 40, 50–52], decreased the magnitude of lytic cycle induction while causing enhanced cell death in EBV-positive NPC cells [53]. We are thus curious to know if these novel inducers could cooperate with the known inducers to induce lytic cycle. We tested various combinations of concentrations of E11 and C7 with the HDAC inhibitor romidepsin and SAHA in AGS-BX1 cells, and found that both HDAC inhibitors could cooperate with E11 and C7 to induce EBV lytic cycle (Fig 6 and S6 Fig). For example, 2.5nM romidepsin, together with low doses (from 2.5 to 10μM of E11 or 1.25 to 2.5μM of C7) of E11 and C7, synergistically induced the expression of the viral IE protein Zta (Fig 6). Similarly, 2.5μM of SAHA could also synergise with 2.5 to 10μM of E11 or 1.25 and 2.5μM of C7 to induce the expression of Zta (S6 Fig).

## Discussion

Although EBV lytic reactivation has been implied in the pathogenesis of EBV-associated malignancies such as NPC and EBV-associated lymphoproliferative diseases [15–18], it is also part of the novel targeted therapeutic strategy–lytic induction therapy–which wakes up the dormant EBV in associated cancer cells to render them susceptible to antiviral treatment [19, 23, 24]. Yet currently the *in vivo* mechanism leading to of EBV reactivation remains largely elusive, despite the demonstration of terminal differentiation of B cells [54] and differentiation of epithelial cells being associated to induction of lytic cycle [46, 55]. The *in vitro* models of reactivation have been better characterised with the help of chemical and physiological reagents that serve as stimuli to trigger the start of the lytic cascade. Nevertheless, the major classes of chemical EBV lytic inducers reported thus far are mainly HDAC inhibitors and phorbol esters [10, 11, 13, 14]. The possibility of other chemical classes to induce lytic cycle in latently-infected EBV cells has remained largely unexplored, with only one screening of a library of 2700 FDA-approved drugs identifying the proteasome inhibitor bortezomib as a strong EBV lytic inducer in B lymphoma cells [50, 51], and a recent study reported a new group of lytic inducers of the tetrahydrocarboline class [56]. At the same time, by far most lytic inducers are cell line-dependent [10, 25–28], which limited their potential to be developed into clinically useful agents. Our study, thus, expands the pool of chemical EBV lytic inducers through our identification of chemical compounds of diverse chemical structures.

The compounds for screening are from a chemical library of 50,240 drug-like molecules with MW ranging from 300 to 700. Contrary to the previously reported HTS to screen for novel lytic inducers [56], the 5 hit compounds we obtained in our screening were of diverse structures, with the exception of compound C7 and C8, both with a hydrazone bridge, these compounds do not seem to resemble each other in terms of structure, thus not possibly belonging to the same chemical family. We argue this could represent the diversity of stimuli that can trigger EBV reactivation in nature, and thus each of the identified hit compounds is a worthwhile target for further investigation. In addition, these hit compounds are structurally unrelated to the main classes of lytic inducers, the HDAC inhibitors and phorbol esters (Figs 1E and 5D), suggesting they may possess different bioactivities from these classical EBV lytic inducers. We thus further compared these compounds against HDAC inhibitors and phorbol esters in terms of their biological action. Unlike HDAC inhibitors, these compounds do not cause the hyperacetylation of histone proteins at various treatment concentrations, including the lytic-inducing concentrations. We have also probed for the changes in phosphorylation of PKCδ, the effector molecule that HDAC inhibitors and the phorbol ester TPA act through [8, 14, 23, 33], after the treatment by the hit compounds, yet no significant changes have been observed (Fig 5A and 5B). We thus believe that, these compounds are neither HDAC inhibitors nor PKC agonists. We have also attempted to compare our hits to the hits of the two other reported screenings for EBV lytic inducers, the proteasome inhibitor bortezomib [50, 51] and the tetrahydrocarbolines [56]. All of our five hits are structurally dissimilar to bortezomib and the tetrahydrocarbolines. In terms of biological activities, although bortezomib has been found to induce lytic cycle on EBV-positive B lymphoma cells [50–52], we found that its induction was relatively weaker in EBV-positive epithelial cells, e.g. NPC cells [53]. In contrast, our hit compounds induced lytic cycle strongly in EBV-positive epithelial cells, suggesting difference in the biological action between our hit compounds and bortezomib. Taken together, hits from our screening are novel lytic inducers with distinct structures and mode of action from the classical lytic inducers that warrant further investigation.

In our detailed examination of lytic reactivation by these compounds, we found two compounds that intrigued us. Compound E11 consistently reactivated EBV to full lytic cycle with the expression of late viral proteins e.g. VCA-p18, gp350/220 in AGS, HONE1 background cells with recombinant EBV genomes, as well as in YCCEL1 (S3 Fig), a Korean GC cell line with native EBV genomes. It was able to induce the production of infectious viral particles in AGS-BX1 and HONE1-EBV cells too. In addition, it reactivated a much higher percentage of cells into lytic cycle compared with other compounds in AGS-BX1 cells, raising interest in its mechanism of action. Besides compound E11, compound C7 is of special interest for its ability to reactivate EBV lytic cycle in a diversity of EBV-positive epithelial cancer lines, including the native EBV genome-carrying C666-1, SNU-719 and YCCEL1 cells. We thus investigated these 2 compounds in greater detail. These two compounds acted invariably fast in AGS-BX1 cells, with the expression of viral IE protein, Zta, being detectable as early as 15min post-treatment by both compounds (S2 Fig). Such fast kinetics also distinguish them from the HDAC inhibitors e.g. romidepsin, in which the expression of Zta could only be observed after 12h of treatment on cell lines of the same background [23], hinting the possibility of difference in mode of action of the novel compounds compared to the HDAC inhibitors such as romidepsin. We contemplate the fast action might be mediated through post-translational modification of signaling molecules, and thus examined whether the incubation with E11 and C7 changes the activation of signaling molecules in kinase pathways that have been previously reported to be involved in lytic cycle reactivation. We found that concurrent with the induction of lytic cycle, there was also a sustained activation of JNK and transient activation of p38 MAPK, suggesting possible involvement of them in the induction of lytic cycle by these compounds. Experiments using the specific pathway blockers to inhibit the relevant pathways indicated that only the JNK inhibitor significantly abrogated the lytic reactivation by E11, and both the MEK inhibitor and JNK inhibitor delivered partial inhibition towards lytic cycle induction by C7. We are aware that caution has to be paid while interpreting data from the use of chemical inhibitors and thus we reckon that further confirmation is required to confirm the role of JNK and/or ERK pathways in leading to EBV lytic reactivation. Nevertheless, we have noted that rottlerin, the PKCδ inhibitor that has been shown to abrogate lytic cycle induction by several HDAC inhibitors, failed to significantly reduce lytic cycle induction E11 and C7, further supporting the notion that these two compounds possess distinct mode of action from the HDAC inhibitors. Although suppression of lytic induction could be observed for some inhibitors for the two compounds, such suppression was only partial, indicating the possibility that more than one pathway might be activated by each compound and those pathways act in concert to bring about the activation of Zta and Rta transcription to start lytic cycle. We thus think that it would be worthwhile to further investigate the mechanisms in which these new compounds activate EBV lytic cycle, such that potential new pathways or new mode of action, could be uncovered.

Combination of sodium butyrate, an HDAC inhibitor, and TPA, a PKC agonist, can synergistically induce lytic cycle in a number of cells [10, 57]. This is used as a strategy to increase the responsiveness of cells towards lytic induction. However, other combinations of lytic inducing stimuli may not induce lytic cycle synergistically, instead, they counteract each other. For example, combination of bortezomib, a proteasome inhibitor which could weakly induce lytic cycle, and SAHA, an HDAC inhibitor, reduced the lytic induction strength although synergistic killing of cancer cells could be achieved [53]. We thus tried to combine our newly identified compounds with HDAC inhibitors extensively studied in our laboratory, romidepsin and SAHA. Interestingly, enhanced induction of lytic cycle was the most obvious at concentrations where each of the agents did not induce lytic cycle strongly. Thus by combining the novel compounds and an HDAC inhibitor, we could lower the concentration of either agent to 1/2 or even 1/4 of the concentration when they were used as single agents to achieve the same or even higher level of lytic induction. This offers an attractive potential to improve the responsiveness of cells to lytic induction therapy, with the compounds serving as leads for further investigation and development.

**Fig 7:**
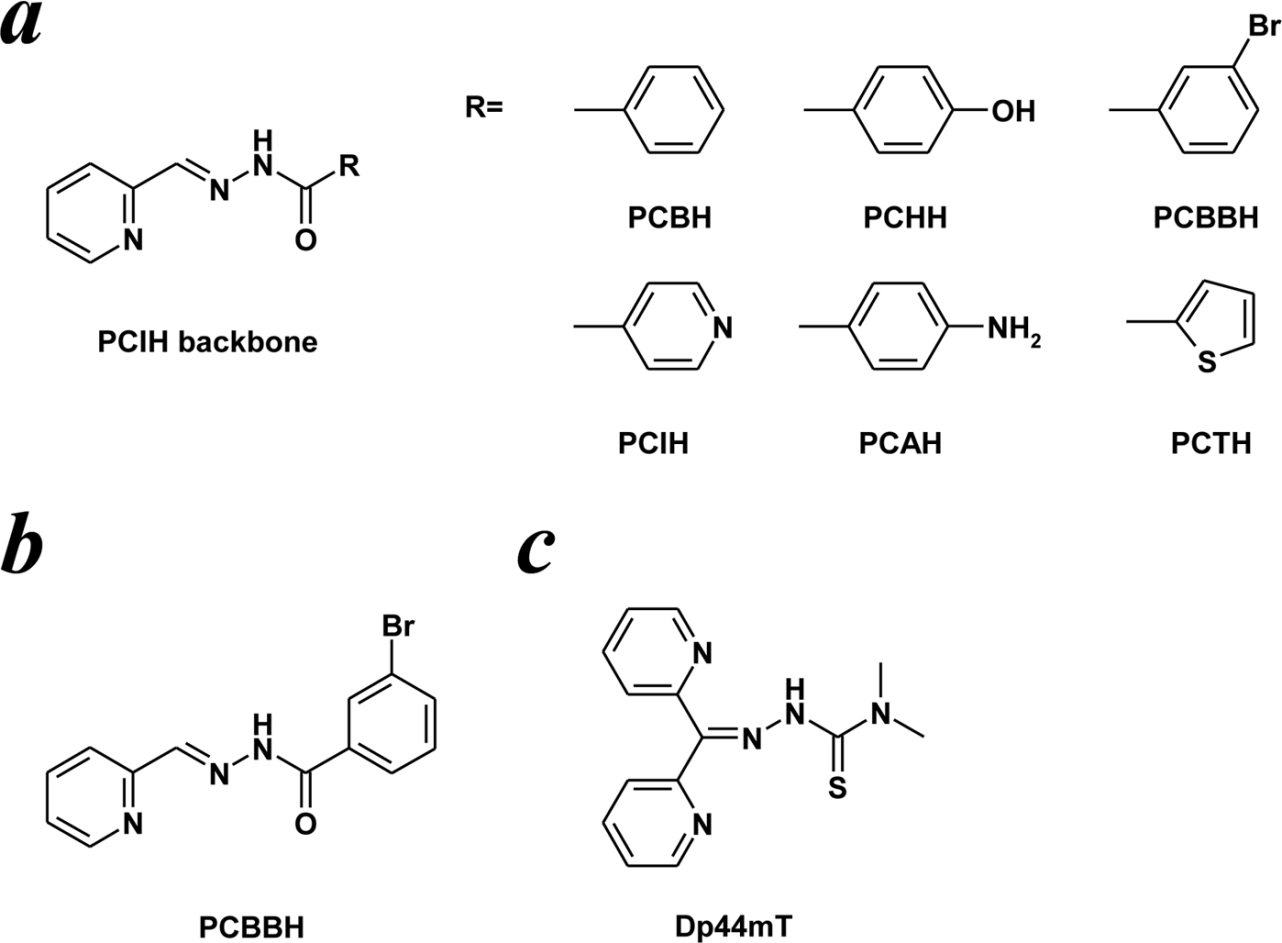
Structures of iron chelators with structural similarity to C7. (a) The structure of the iron chelator 2-pyridylcarboxaldehyde isonicotyinoyl hydrazone (PCIH) and its analogues. (b) The structure of 2-pyridylcarboxaldehyde *m*-bromobenzoyl hydrazone (PCBBH), the iron chelator in the PCIH series in which C7 is most structurally similar to. (c) The structure of di-2-pyridylketone-4,4-dimethyl-3-thiosemicarbazone (Dp44mT), a well-known iron chelator.

Owing to the ability of C7 to reactivate EBV lytic cycle in all the cell lines tested, we investigated in greater detail if its structure displays similarity to any groups of known compounds or drugs. The hydrazone bridge of C7 is a common structural entity among synthetic iron chelators, e.g. 2-pyridylcarboxaldehyde isonicotinoyl hydrazone (PCIH) and its analogs [58], and very similar to the well-known iron chelator di-2-pyridylketone-4,4-Dimethyl-3-thiosemicarbazone (Dp44mT) (Fig 7A and 7C). Among them, compound C7 bears the greatest resemblance to the 2-pyridylcarboxaldehyde m-bromobenzoyl hydrazone (PCBBH) (Fig 7B). It is very likely that C7 also possess iron-binding or iron-chelating capabilities like PCBBH and Dp44mT. As iron chelators have not been previously reported in research studies to induce EBV lytic cycle in EBV-associated epithelial cancers, we reckon that further investigation into this would open up a possibility to a new large class of chemicals that induce EBV lytic cycle in EBV latently-infected cells.

In conclusion, our screening identified 5 novel organic compounds as EBV lytic inducers. Their diverse chemical structures and different biological action from classical EBV lytic inducers, e.g. HDAC inhibitors and TPA, make them attractive targets for further investigation. We investigated 2 of the 5 compounds in greater detail, and found that both displayed fast lytic inducting kinetics. One of them is a potent activator of the MAPK pathways, especially the JNK pathway, while the other is structurally similar to iron chelators. While more efforts are required to delineate the exact mechanism of action, these compounds represent good candidates of new class(es) of EBV lytic inducing stimuli, new chemical tools to better decipher alternative mechanisms leading to activation of lytic cycle in latently-infected cells, and lead compounds for further development to be used in conjunction with current lytic inducers to improve responsiveness of cells to lytic induction.

## Supporting Information

S1 Fig Tertiary screening in NA cells. (a) Expression of EBV immediately-early (IE) lytic proteins, Zta, Rta, and early protein EA-D (BMRF1) in NA cells 48h post-treatment by the top 40 compounds in tertiary screening. The concentrations used were the approximate half inhibitory concentration (IC_50_) for cell proliferation. The 22 compounds with an asterisk (*) below their code were selected for further comparison of lytic protein expression and viral genome replication upon addition to the cells. (b) & (c) Expression of EBV IE and early proteins and replication of viral genome 48h post-treatment induced by the selected 22 compounds on NA cells.
(TIF)

S2 Fig Lytic induction kinetics of the hit compounds at early periods of treatment. AGS-BX1 cells were treated with the hit compounds at various time points to observe for the time point in which increase in expression of the viral IE protein Zta was first detected. Compound E11 and C7 is the fastest to induce lytic cycle, with the increase in Zta expression first detected at 0.25h, i.e. 15min post-treatment.
(TIF)

S3 Fig Expression of various lytic proteins induced by the hit compounds in HONE1-EBV and YCCEL1 cells. HONE1-EBV cells or YCCEL1 cells were treated with the hit compounds at their optimal concentration to induce lytic cycle. The expression of various EBV lytic proteins was detected at different time points post-treatment. Compound E11 consistently induced the expression of late proteins (e.g. p18-VCA) in cell lines it is capable of inducing lytic cycle.
(TIF)

S4 Fig Activation of the cellular kinase pathways by romidepsin and compound E11. AGS-BX1 cells were treated with romidepsin (R) at 5nM for 24h or E11 at 20μM at the specified time points. Romidepsin treatment increased phosphorylation of PKCδ and ATM but not JNK, while vice versa for E11.
(TIF)

S5 Fig Rotterlin, a specific PKCδ inhibitor, inhibited lytic induction by the HDAC inhibitor SAHA. HONE1-EBV cells were pre-treated with specific inhibitors of PI3K (LY294002, 15 μM), MEK (PD98059, 50μM), JNK (SP600125, 50μM), p38 MAPK (SB202190, 20μM) and PKCδ (Rottlerin, 10μM) for 1h before the addition of 10μM SAHA. Cells were harvest after 48h for examination of lytic induction by western blotting. Only rottlerin significantly hampered lytic induction by SAHA in HONE1-EBV cells.
(TIF)

S6 Fig Enhanced induction of EBV lytic cycle by the hit compounds and the HDAC inhibitor SAHA. AGS-BX1 cells were treated with 2.5μM of SAHA and various concentrations of E11 or C7 for 24h. Expression of viral IE protein Zta was detected to by western blotting to estimate the magnitude of lytic induction. The combinations with an asterisk (*) are the concentrations at which enhanced induction was observed.
(TIF)

## Abbreviations

EBV: Epstein-Barr virus
HDAC: histone deacetylase
SAHA: suberoylanilide hydroxamic acid
NPC: nasopharyngeal carcinoma
GC: gastric carcinoma
