# Supplementary figures and images for: Identification of Novel Small Organic Compounds with Diverse Structures for the Induction of Epstein-Barr Virus (EBV) Lytic Cycle in EBV-Positive Epithelial Malignancies
Source: PLoS One. 2015 Dec 30;10(12):e0145994. doi: 10.1371/journal.pone.0145994 (PMC4696655; doi:10.1371/journal.pone.0145994)

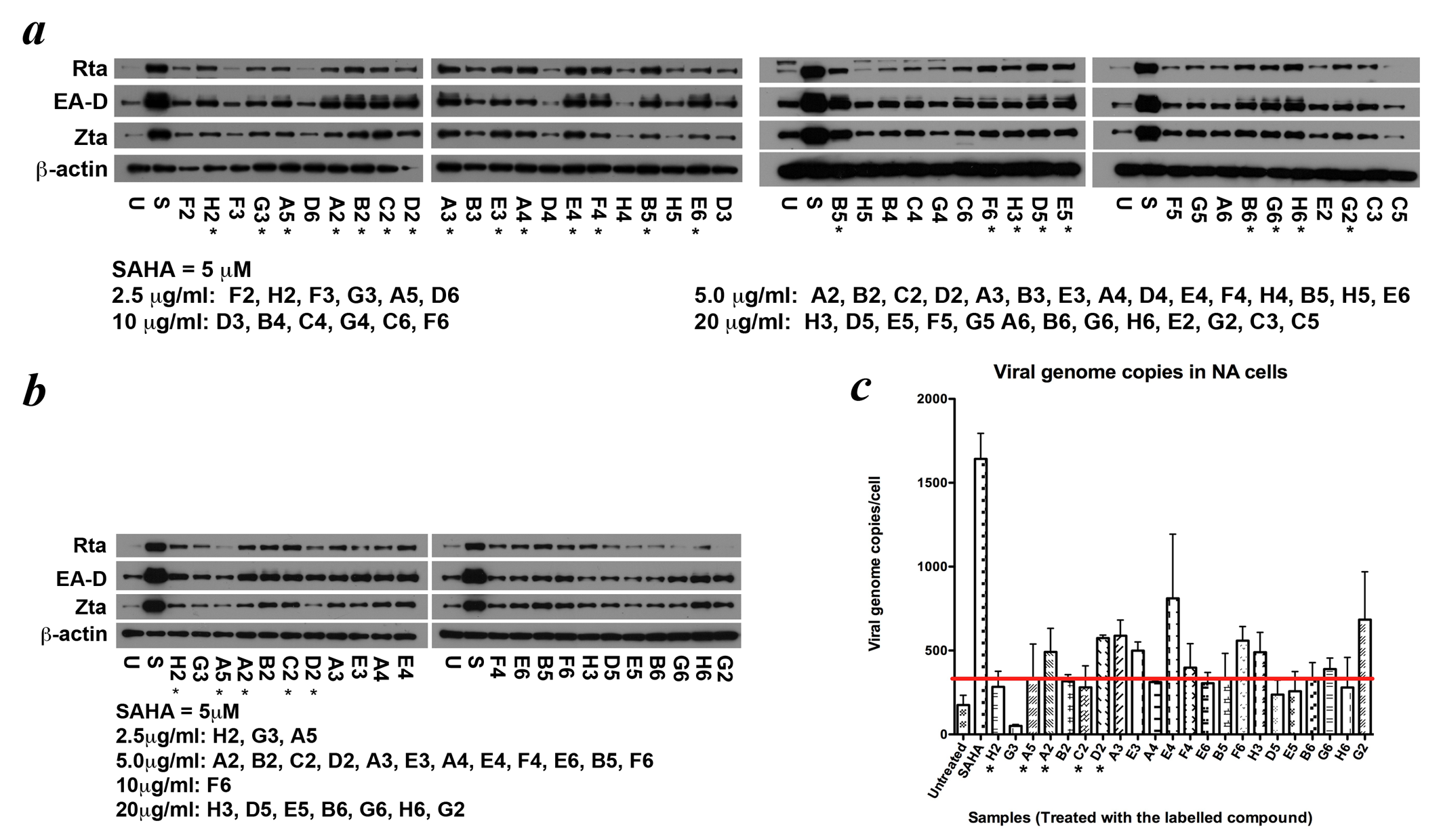

Supplement: S1 Fig — (a) Expression of EBV immediately-early (IE) lytic proteins, Zta, Rta, and early protein EA-D (BMRF1) in NA cells 48h post-treatment by the top 40 compounds in tertiary screening. The concentrations used were the approximate half inhibitory concentration (IC50) for cell proliferation. The 22 compounds with an asterisk (*) below their code were selected for further comparison of lytic protein expression and viral genome replication upon addition to the cells. (b) & (c) Expression of EBV IE and early proteins and replication of viral genome 48h post-treatment induced by the selected 22 compounds on NA cells. (TIF) [file pone.0145994.s001.tif]

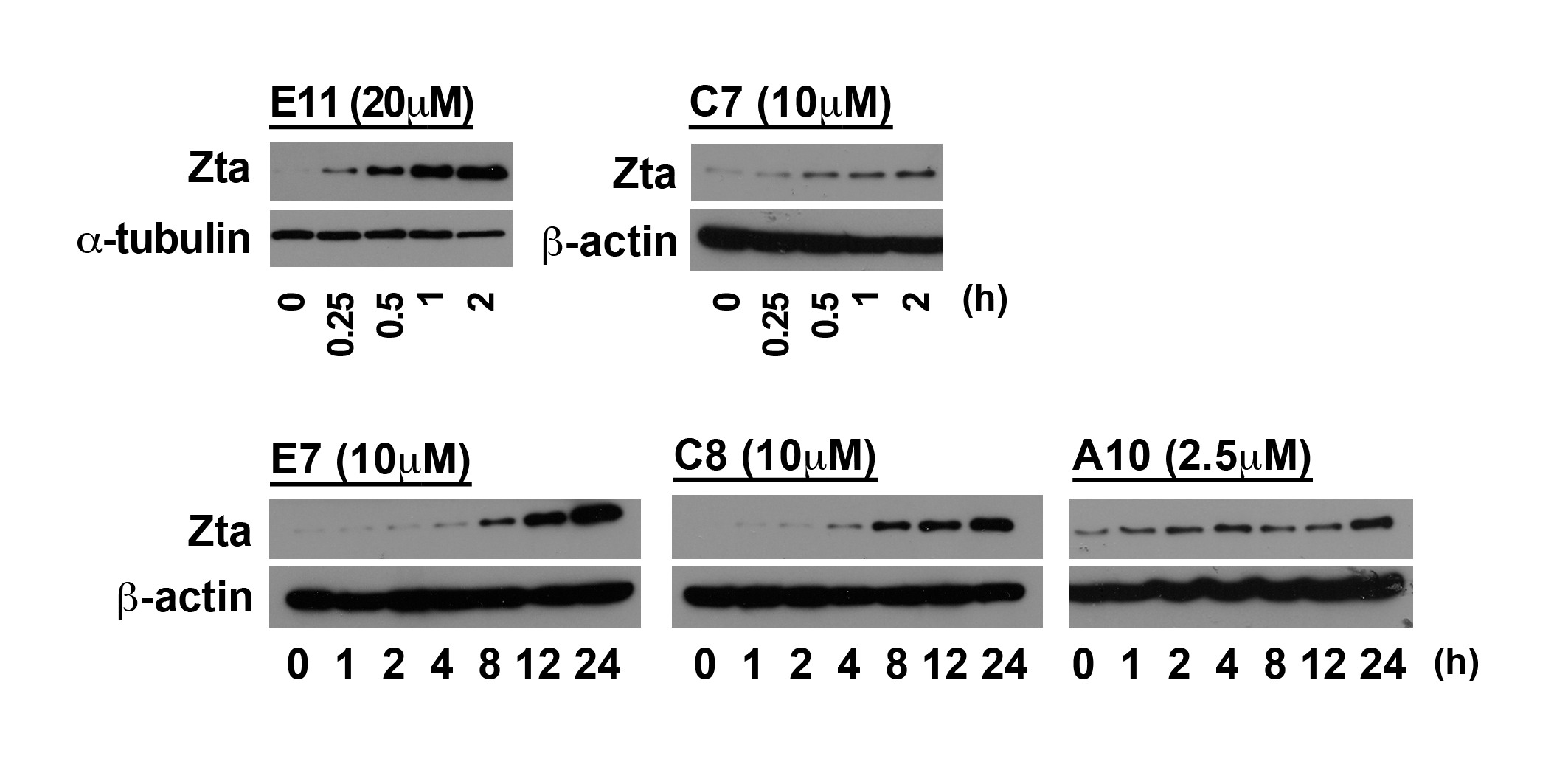

Supplement: S2 Fig — AGS-BX1 cells were treated with the hit compounds at various time points to observe for the time point in which increase in expression of the viral IE protein Zta was first detected. Compound E11 and C7 is the fastest to induce lytic cycle, with the increase in Zta expression first detected at 0.25h, i.e. 15min post-treatment. (TIF) [file pone.0145994.s002.tif]

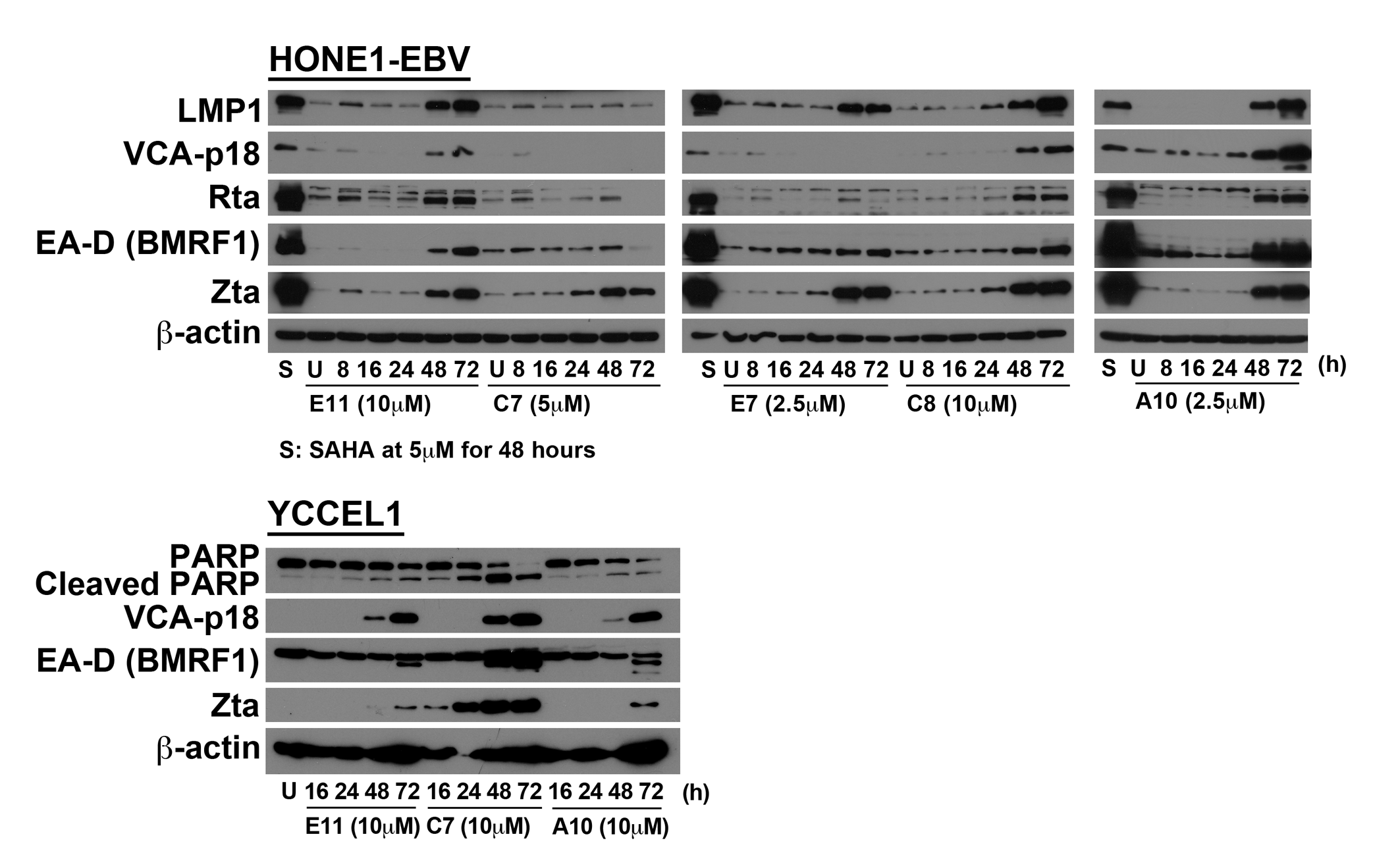

Supplement: S3 Fig — HONE1-EBV cells or YCCEL1 cells were treated with the hit compounds at their optimal concentration to induce lytic cycle. The expression of various EBV lytic proteins was detected at different time points post-treatment. Compound E11 consistently induced the expression of late proteins (e.g. p18-VCA) in cell lines it is capable of inducing lytic cycle. (TIF) [file pone.0145994.s003.tif]

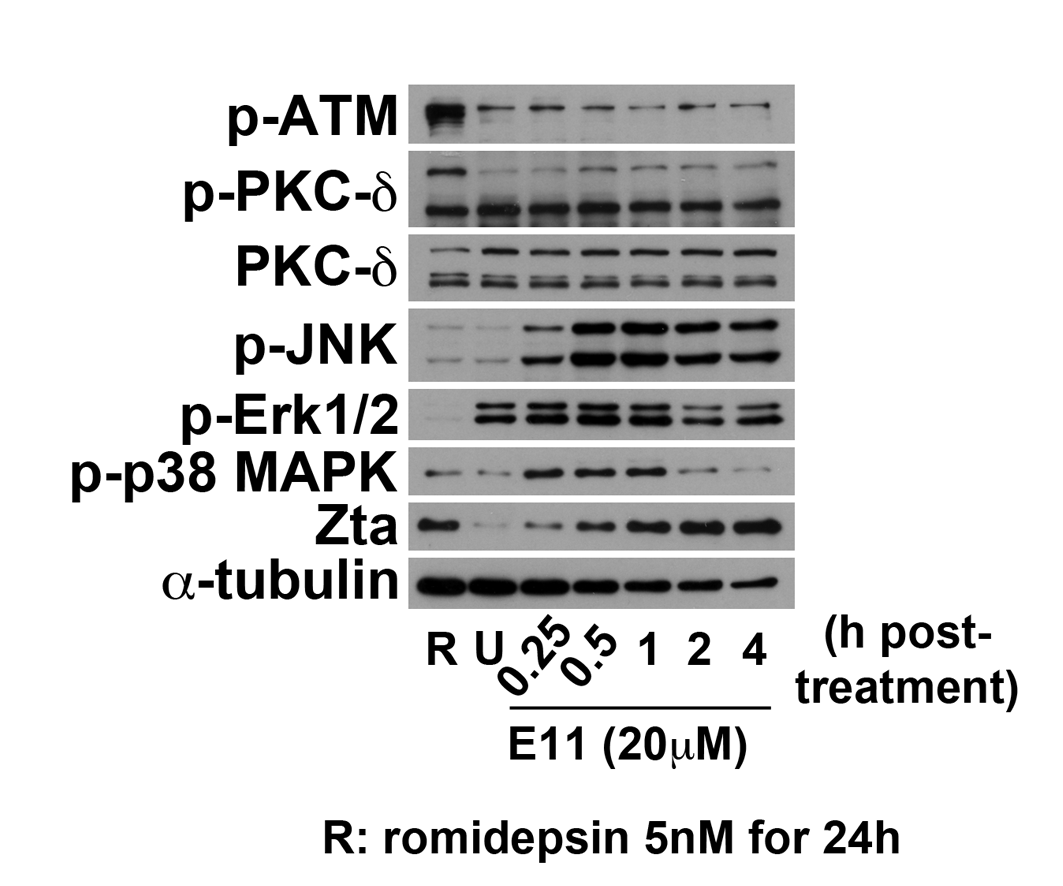

Supplement: S4 Fig — AGS-BX1 cells were treated with romidepsin (R) at 5nM for 24h or E11 at 20μM at the specified time points. Romidepsin treatment increased phosphorylation of PKCδ and ATM but not JNK, while vice versa for E11. (TIF) [file pone.0145994.s004.tif]

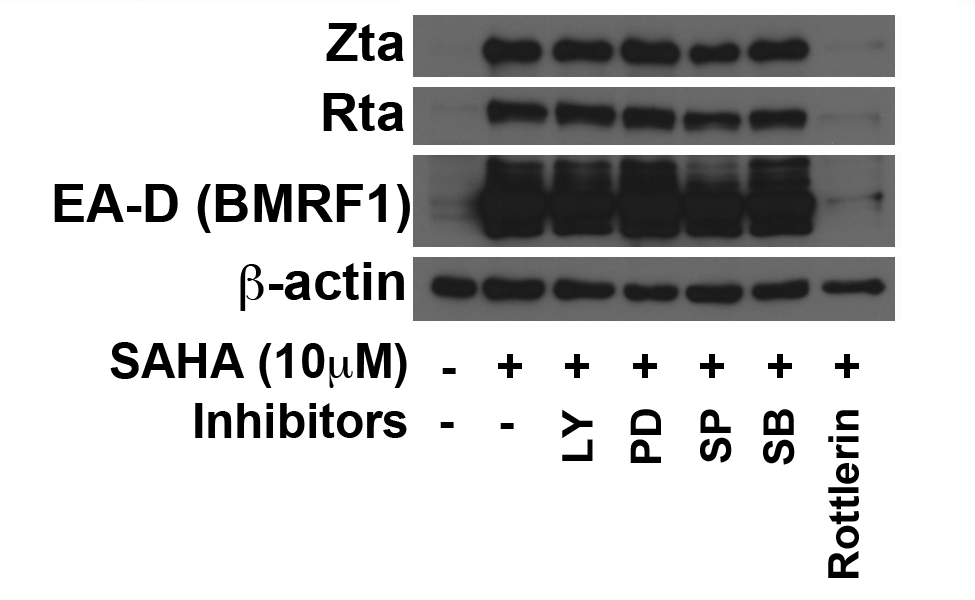

Supplement: S5 Fig — HONE1-EBV cells were pre-treated with specific inhibitors of PI3K (LY294002, 15 μM), MEK (PD98059, 50μM), JNK (SP600125, 50μM), p38 MAPK (SB202190, 20μM) and PKCδ (Rottlerin, 10μM) for 1h before the addition of 10μM SAHA. Cells were harvest after 48h for examination of lytic induction by western blotting. Only rottlerin significantly hampered lytic induction by SAHA in HONE1-EBV cells. (TIF) [file pone.0145994.s005.tif]

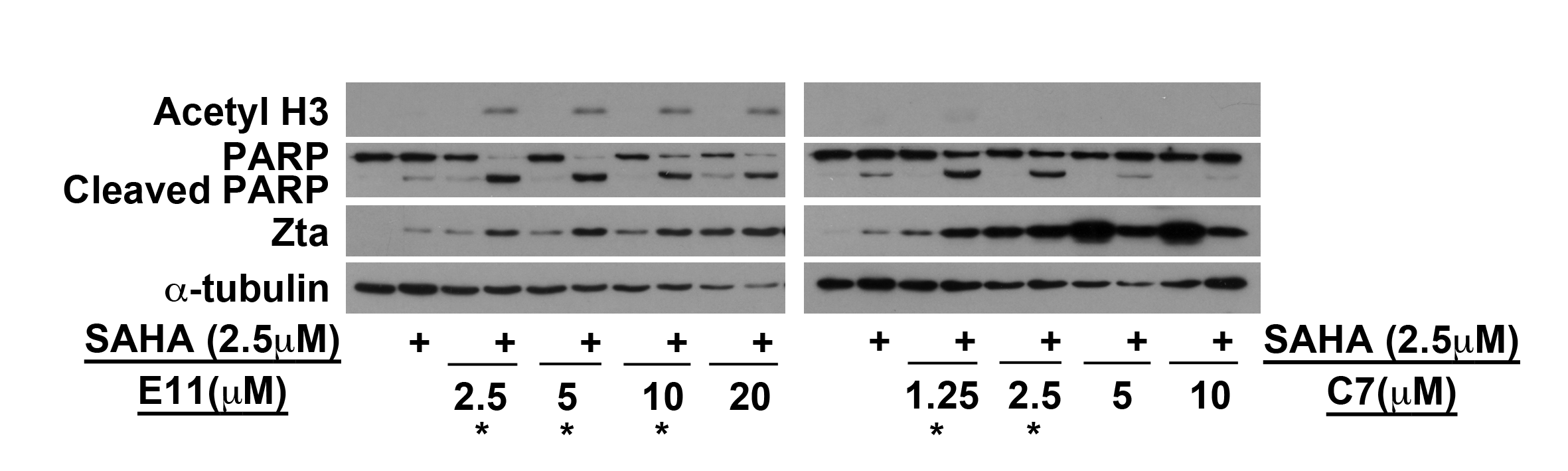

Supplement: S6 Fig — AGS-BX1 cells were treated with 2.5μM of SAHA and various concentrations of E11 or C7 for 24h. Expression of viral IE protein Zta was detected to by western blotting to estimate the magnitude of lytic induction. The combinations with an asterisk (*) are the concentrations at which enhanced induction was observed. (TIF) [file pone.0145994.s006.tif]
